# Poisoning of Cattle by Andropogon Sorghum

**Published:** 1897-11

**Authors:** H. T. Pease

**Affiliations:** Principal Veterinary College, Lahore, India


### THE JOURNAL
OF
### COMPARATIVE MEDICINE AND VETERINARY ARCHIVES.

VOL. XVIII. *NOVEMBER, 1897.* No. 11.

### POISONING OF CATTLE BY ANDROPOGON SORGHUM.

BY VETERINARY CAPTAIN H. T. PEASE, F.Z.S., F.R.M.S.,
PRINCIPAL VETERINARY COLLEGE, LAHORE, INDIA.

ANDROPOGON sorghum is a tall, handsome grass extensively cultivated throughout India. As a fodder it may be given green, or after having been dried and preserved for months. Some varieties are especially grown as cattle-fodder, in which case the stalks are cut when green, before the seed matures. It is stacked, and forms in some parts of the country the chief food of the cattle during the months when pasture is not to be had. It is very nutritious, and cattle do well on it. The seed is eaten by the people, and the grain holds, in India, a position very much like that of oats in Scotland. Indeed, by the natives it is regarded as more wholesome than wheat or rice, being more easily digested. The seed contains, normally, Professor Church informs us, 21 per cent. of potash.

It has for a long time been known to the native agriculturists, and to others who take an interest in cattle in India, that the usually wholesome and useful fodder-plant, "juar," andropogon sorghum, becomes, in certain circumstances, poisonous, and causes the death of animals which eat it in any quantity.

The conditions in which the plant becomes harmful are, when, owing to the failure of the rains, the plant becomes stunted and dried up. The popular idea among the natives of this country is that, in these conditions, the plant becomes attacked by a small insect to which they give the name of "bhaunri," and it is to the animal eating this insect with the juar that they attribute death. The disease is known to the people of the Punjab, where it is common, as "pathalargaya" or "pathalaggaya."

The fact of animals being poisoned by this plant has given rise, at various times, to some inquiries being made as to the cause, and various theories have been advanced to account for it. Some of these may be found quoted at length in the *Dictionary of Economic Products* under the heading "Sorghum." It will be seen that some have adopted the insect-theory. The following passage occurs: "It is otherwise with the insect-pests. Some of them are fully understood, others are so obscure that much difference of opinion prevails as to whether the poisonous property spoken of as possessed by the stems at times, when used for fodder, is due or not to an insect." In the special article on "Pests," in the same dictionary, it is stated "that the larvæ of a moth known in the northwestern provinces as bhaunri attack the juar-stalks in much the same way as the sugar-cane is channelled by the sugar-cane borer. These larvæ, in fact, bear so strong a resemblance to those found in the cane that Mr. Cotes suggests that they may set up decomposition sufficient to cause the poisonous properties of juar, regarding which so much has been written. It may be added that the prevalent idea among the natives is that the poison is the result of an insect, and it is worthy of note that it occurs at the same period and under similar conditions as in sugar-cane. The two following passages may be taken as representing the somewhat exhaustive controversy that exists on this subject: "The most peculiar disease to which juar is liable is that which makes the stalks poisonous to cattle if eaten by them when semi-parched from want of rain. Of the fact there can be little doubt. In the scarcity of 1877 great numbers of cattle were known to perish from this cause, their bodies becoming inflated after a meal on the young juar-plants, and death ensuing shortly afterward, apparently in severe pain. A good explanation, however, is not forthcoming. The opinion which is usually accepted by the natives is that young sorghum, when suffering from deficiency of rain, becomes infected by the insect to which its poisonous effects on cattle are due. Immediately rain falls the insect is said to perish, and unless the ears have appeared before the rain failed the crop often recovers itself and yields a good outturn of grain.

A totally different explanation of the great mortality among cattle in the year 1887 is given in a paper by Veterinary Surgeon J. Anderson (*Agricultural and Horticultural Society Journal*, new series). The following extracts will show the opinions arrived at by that writer: "From my recent experiments and investigation I have come to the conclusion that juar is not poisonous. Some stalks here and there contain insects, others a fungus, both of which are supposed by many to be the medium of the poison. Even if they were poisonous, they are not found in sufficient quantity to prove injurious and account for such wholesale mortality. The prevailing idea is that the plant has become poisonous not only from want of the usual rains, but also from the effect of the unusually hot winds, or that a poisonous gas is engendered in the stalks by the heat, etc. I look upon the plant as a destructive substance or thing, and not as a poison. It destroys life by acting mechanically on the system." The writer is of opinion that the disease is nothing more nor less than the ordinary tympanitis in the epidemic form. This explanation cannot be regarded as a satisfactory one, for the plant is only injurious in certain conditions, and loses its harmful properties completely after rain has fallen. I think, therefore, that we are justified in searching further for the cause of the excessive mortality induced by the plant.

The conditions under which sorghum andropogon becomes injurious are climatic disturbances, such as want of rain, excess of humidity, or damp, cloudy weather and extreme and unnaturally high temperature. It will be observed from the remarks already offered as to whether it is due to the presence of an insect or to some physiological changes in the growth of the plant, owing to climatic disturbances, the stems are not always liable to cause injury to cattle. The occurrence of the poisonous property, as it has been called, is simultaneous over a large tract of country, appearing and disappearing within certain fixed limits of time. The inference is, therefore, unavoidable that the stems have been affected or altered in some way.

The plant is generally grown on high lands as a "pharif" crop, and is only occasionally irrigated. It is dependent on the rains for its moisture. During my tour of last year in the Punjab I found mentioned in all the villages visited that this was a very fatal form of poisoning, the nature of which I could only speculate upon, as I was unable to see any cases of it at that season. Owing to the failure of the rains and the prevalence of a high temperature in 1895, however, I have been able to see animals which have succumbed as well as those the subjects of disease. Certain points at once struck me in connection with them. It appeared that the plant only gave rise to the symptoms when it had been stunted and withered up, when it had grown to a certain height, and when the rains had failed. Another point noticed was the rapidity with which the symptoms developed and led to a fatal termination, death being almost apoplectiform in some cases, and the majority of the animals attacked dying very rapidly. The people informed me that a crop of the plant which had become stunted and poisonous lost its poisonous properties if heavy rain fell. Taking these points into consideration, and looking to the symptoms shown by the affected animals, I came to the conclusion that the deaths might be ascribed to the action of an irritant poison giving rise to gastro-enteritis, and causing marked alterations in the blood. On inspection of the juar which had been given to the dead animals I was very much surprised on breaking open the dried stalks to find a considerable quantity of a white salt deposited in crystals in the pith, more especially at the nodes. The salt, to the taste, was cooling and saline, and on burning a piece of the stalk there was marked crepitation. I collected some of the stalks and subjected them to a chemical examination, which revealed the fact that the salt was nitrate of potash. The quantity of the salt in the stem was so considerable that there was little doubt in my mind that this was the cause of the death of the cattle which had been fed upon it.

At the last Sirsa cattle-fair a number of deaths were caused by poisonous sorghum, and the veterinarian to whom I had mentioned the fact of animals having been killed by the development of nitrate of potash in the stems found the plant in the same condition as that which I had previously examined. He sent me a small bundle of it for opinion, and I took the opportunity of having it carefully analyzed by Thomas Stephenson, a properly qualified analytical chemist, in Bombay, who reports as follows:

"I have made a chemical analysis of a sample of andropogon sorghum, and find that the stem contains nitrate of potash to the extent of 75 grains per ounce weight of the plant. This proportion refers to the bulk sample given to me by Veterinarian Captain Pease, but the salt is very unevenly distributed throughout the plant, being most abundant in the stem at the nodes or junction of the leaves."

Judging from these observations, I have no doubt whatever that the sorghum-plant, at times and in certain circumstances, such as have been mentioned above, becomes distinctly poisonous, owing to the amount of nitrate of potash which is contained in the stems.

The symptoms of poisonous doses of nitrate of potash are gastro-enteritis, tympanites, nausea and colic, staggering gait, stupefaction, polyuria, and death. Authorities on the subject state, sometimes, death from nitrate-of-potash poisoning is apoplectiform, and we may at times observe spasms and rotation of the eyeballs.

It is usually very rapid; the animal may succumb in a few minutes. Usually it lasts about half an hour, but seldom over twelve hours. The post-mortem appearances are those of hemorrhagic gastro-enteritis, cherry-red or purple coloration of the gastric mucous membrane and that of the small intestine, and superficial ulceration of this membrane. The intestinal contents are reddish-brown. The kidneys are ecchymosed and inflamed. The intensity of the effects of the drug are in inverse ratio to the fulness of the stomach. There can be no reasonable doubt that in some cases of poisoning from the sorghum-plant the cause is the presence of large quantities of nitrate of potash in the stems. Some of the samples I have examined contain 25 per cent. of this salt, so that a very small feed of them would introduce sufficient to carry off the animals which ate them.

I have been much interested to find that Veterinary Surgeon Mayo related similar experiences at the meeting of the United States Veterinary Medical Association, held in September, 1896, and published in the JOURNAL OF COMPARATIVE MEDICINE for June, 1897. Mr. Mayo, in the course of his remarks, is reported as follows: "Two years ago seven out of twelve head of cattle died during one night from the effects of potassium nitrate which was contained in four armfuls of cornstalks. These cornstalks had been grown upon very rich soil, and a large amount of potassium nitrate—25 per cent. of the dry weight of the stalks in some instances—had been stored within or deposited upon the cornstalk. It was then thought that poisonous quantities of potassium nitrate would only be found in corn grown on soil which had been heavily manured; but since then I have found potash present in poisonous quantities in cornstalks grown without fertilizer upon bottom and upland soils. Less than eight ounces of the salt will produce fatal effects in adult cattle. Poisonous quantities of potassium nitrate in the plant often occurs in patches not recognized by any noticeable difference in the growth of the corn. The presence of potash in poisonous quantities in the cornplant may be influenced by climatic conditions. In this State there is often a period of dry weather which may check the metabolic process in the plant and thus favor the deposition of potassium nitrate." It is curious to note that this peculiarity occurs in several species of the sorghums, and we find *S. hallpense* (baru) also poisonous in certain circumstances. As this plant is often the only one available for cattle-food in seasons of scarcity, owing to want of rain, it has been suggested that cutting up and washing will deprive it of its fatal properties so that it may be used as fodder.

